# Is Less More? Limited Surgery Is Insufficient in the Treatment of Spinal Hydatid Cysts

**DOI:** 10.3390/jcm14186540

**Published:** 2025-09-17

**Authors:** Mustafa Emre Sarac, Zeki Boga, Semih Kivanc Olguner, Ali Arslan, Ahmet Hamit Çınkı, Mehmet Ozer, Yurdal Gezercan

**Affiliations:** Department of Neurosurgery, Adana City Traininig and Research Hospital, Adana 01230, Turkey; zekiboga2013@gmail.com (Z.B.); kivanc3olguner@hotmail.com (S.K.O.); aliarslan26062006@hotmail.com (A.A.); cinkiahmet@gmail.com (A.H.Ç.); mehmetozerdr@gmail.com (M.O.); gezercan@hotmail.com (Y.G.)

**Keywords:** echinococcosis, spinal diseases, spondylectomy, laminectomy, recurrence

## Abstract

**Background/Objectives**: Spinal hydatid disease frequently poses significant surgical challenges and leads to severe neurological complications. Despite the development of various surgical techniques, recurrence remains a common issue. The aim of this study was to evaluate the impacts of radical vertebrectomy on recurrence and long-term follow-up outcomes by comparing total en-bloc spondylectomy with conventional laminectomy, decompression, and posterior stabilisation in patients treated at our centre. **Methods**: This study included 21 patients who underwent surgery for spinal hydatid cysts at our centre between 2001 and 2021. Twelve patients had cystectomy, laminectomy, decompression, and stabilisation, and nine patients had total en-bloc spondylectomy. A single senior surgeon carried out each procedure, selecting the surgical approach based on the presence of vertebral body involvement. All patients received albendazole treatment for six months following surgery. The surgeon who performed the operations followed up all patients clinically and radiologically for at least three years. **Results:** Seven out of twelve patients (58.3%) who had conventional surgery experienced recurrences, while total en-bloc spondylectomy produced no recurrences (*p* = 0.004). The recurrent cases had a mean of 2.8 surgical procedures and manifested within 14 months. Although total en-bloc spondylectomy was associated with a longer operative time and greater blood loss, neurological recovery and overall clinical outcomes were comparable between the two groups. The difference in the recurrence rate was statistically significant. **Conclusions**: Although technically demanding, radical vertebrectomy is shown to provide complete protection against recurrence in appropriately selected patients with spinal hydatid disease in this study. Furthermore, as conservative approaches often require multiple procedures, total en-bloc spondylectomy can be considered an effective treatment for patients with vertebral body involvement.

## 1. Introduction

Hydatid cyst disease is a parasitic infection caused by *Echinococcus granulosus*, predominantly affecting communities engaged in animal husbandry [1]. This disease is endemic in the Mediterranean basin, the Middle East, Central Asia, South America, Australia, and Western China, and its high prevalence is linked to widespread sheep farming, close contact with dogs, and poor hygiene practices [2].

Dogs serve as the definitive host, while humans are intermediate hosts. The parasite’s eggs are ingested through contaminated food or water. The liver (60–70%) and lungs (20–30%) are the most commonly affected organs, while the spleen, kidneys, brain, and skeletal system are less frequently involved [3]. Bone involvement occurs in 0.5–4% of cases, with half affecting the spine [4,5]. In spinal cases, recurrence rates of 30–100% have been reported [1,4,6]. Recurrence can lead to significant disability, prolonged hospitalisation, and high treatment costs [7].

The thoracic spine is the most frequently affected area, followed by the lumbar, sacral, and cervical regions [7]. The larvae invade cancellous bone tissue, resulting in multivesicular lesions [8,9]. The expansion of cysts into dural or extradural spaces causes the compression of neural structures, resulting in spinal cord injury and further neurological decline [10,11].

Magnetic resonance imaging (MRI) is the most sensitive diagnostic tool [3]. T2-weighted sequences show hyperintense lesions, and T1-weighted sequences show hypointense signals. The peripheral rim enhancement appears in contrast-enhanced scans. Computed tomography (CT) is superior for assessing bone destruction [8]. Serological tests, such as IgG-ELISA, have a sensitivity of 60–90% [3].

Surgical cyst excision combined with albendazole remains the mainstay of treatment [7,12]. There is no consensus regarding the optimal surgical technique, and most studies have been limited to small case series [1]. The impacts of different surgical methods on recurrence have not yet been fully established [3], and long-term outcomes remain insufficiently characterised [5].

Total en-bloc spondylectomy (TES) has been recorded alongside cystectomy, laminectomy, decompression, and posterior stabilisation (CLDS) [13,14]. The effect of TES on recurrence is not fully understood, but the complete removal of parasite-infested vertebral structures may reduce the risk of recurrence [2]. The objective of this study is to compare recurrence and long-term outcomes between TES and CLDS in patients with spinal hydatid disease. The clinical dilemma is whether limited decompression and stabilisation (CLDS) are sufficient, or whether radical total en-bloc spondylectomy (TES) provides superior recurrence prevention.

## 2. Materials and Methods

### 2.1. Study Design and Patient Selection

This single-centre retrospective cohort study included patients who underwent surgical treatment for spinal hydatid cyst disease at the Adana City Training and Research Hospital Neurosurgery Clinic between 1 January 2001 and 30 September 2021. All surgeries were performed by the same senior neurosurgeon. The inclusion criteria were being 18 years of age or older; having a histopathologically confirmed diagnosis of hydatid cyst disease; having a lesion located in the thoracic, thoracolumbar, or lumbar spine; having undergone primary surgery performed using one of two defined surgical techniques (TES or CLDS); having been followed up for at least 36 months; and having undergone at least 6 months of albendazole treatment postoperatively. Cases with cervical or sacral lesions, patients who did not attend regular follow-up appointments, and those who died during the follow-up period were excluded from the study.

Histopathological confirmation was performed intraoperatively from the resected cyst wall and cyst contents, with standard microscopic evaluation by pathology services.

### 2.2. Collection of Clinical and Surgical Data

The demographic and clinical data collected included age, gender, symptom duration, presenting complaints (back pain, motor/sensory deficits, sphincter dysfunction, etc.), pre- and postoperative Frankel scores, lesion location, number of affected vertebrae, involvement of the vertebral body and/or posterior elements, paravertebral spread, spinal canal invasion, operation time, estimated blood loss, transfusion requirement, intraoperative findings (controlled cyst rupture, dura tear, etc.), recurrence, and total follow-up duration. The surgeon’s personal follow-up archives and hospital records provided all of the data.

### 2.3. Radiological and Laboratory Assessments

Peripheral eosinophil ratio, erythrocyte sedimentation rate (ESR), C-reactive protein (CRP), hydatid-specific IgG ELISA, and indirect haemagglutination (IHA) tests were among the preoperative laboratory parameters assessed. Contrast-enhanced spinal MRI was used for radiological follow-ups. Preoperative and postoperative imaging was performed at 3, 6, and 12 months, and then once a year after that. Using a standard evaluation form, the lesion’s location, the extent of spinal canal invasion, and the involvement of the vertebral body and/or posterior elements were noted.

### 2.4. Methods of Surgery

**Figure 1:**
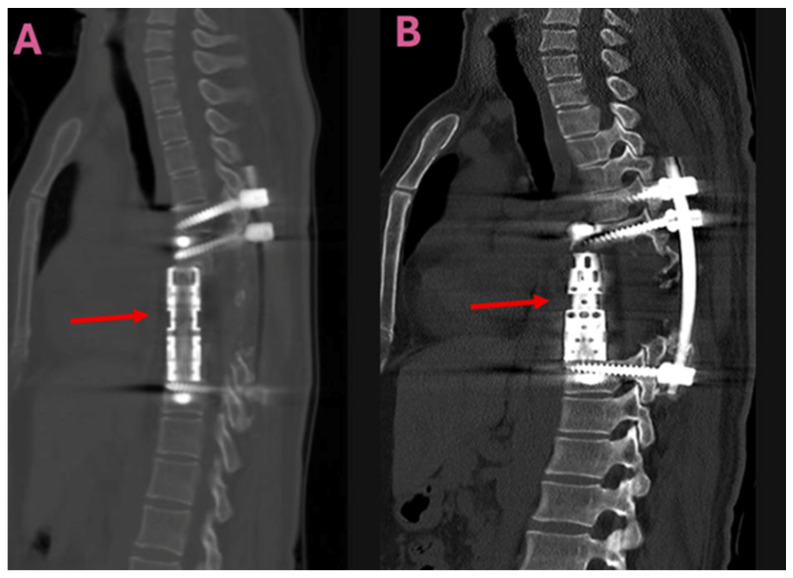
Postoperative sagittal CT images taken following total en-bloc spondylectomy (TES) for a thoracic spinal hydatid cyst. (**A**,**B**): Sagittal CT scans demonstrating vertebral body reconstruction and posterior instrumentation. Arrows indicate the titanium interbody cage used for vertebral body reconstruction. Abbreviations: CT, computed tomography; TES, total en-bloc spondylectomy.

Depending on the surgical method used, the patients were assessed in two groups. Group 1 (TES group, *n* = 9) consisted of patients who underwent total en-bloc spondylectomy, anterior column reconstruction with a titanium cage, and posterior instrumentation covering the adjacent levels (Figure 1).

**Figure 2:**
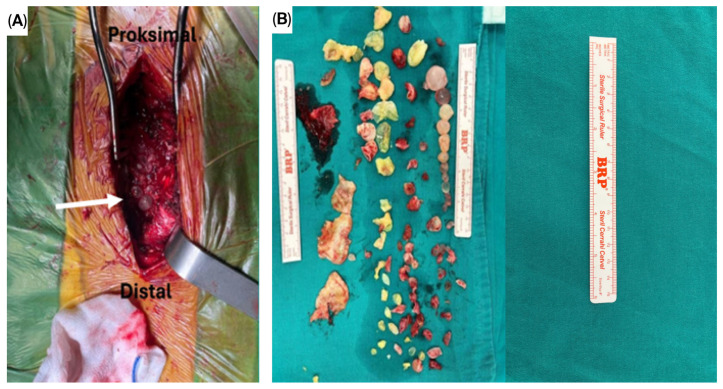
Intraoperative findings in a patient with thoracic spinal hydatid cyst treated with CLDS. (**A**): The intraoperative view shows multiple hydatid cysts occupying the surgical field before cystectomy with an arrow pointing to them. (**B**): The excised cyst materials and vertebral bone fragments which were collected after cystectomy and laminectomy. Abbreviation: CLDS—cystectomy, laminectomy, decompression, and stabilisation.

The patients in Group 2 (CLDS group, *n* = 12) underwent cystectomy, laminectomy, spinal decompression, and posterior stabilisation procedures. The irrigation–aspiration technique was used to drain the cyst contents in both groups when complete removal was not possible before irrigation with 3–5% hypertonic saline to prevent secondary contamination and recurrence. The CLDS method produced intraoperative images which showed that multiple hydatid cysts at the thoracic-spine level completely filled the surgical field before cystectomy while touching the dura and surrounding tissues (Figure 2A). Figure 2B shows the numerous cyst materials and vertebral bone fragments removed after cystectomy and laminectomy.

**Figure 3:**
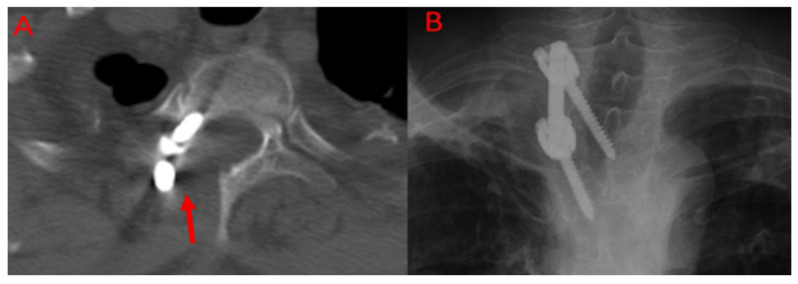
Imaging of a patient with a thoracic spinal hydatid cyst after unilateral CLDS. (**A**): Axial CT image highlighting the laminectomy site and stabilisation hardware used in the unilateral CLDS procedure (the arrow indicates the surgical instruments and laminectomy site). (**B**): Anteroposterior (AP) X-ray of the same patient, demonstrating the posterior instrumentation.

The surgical technique was not randomised, and the selection criteria were determined according to the framework of a surgical protocol that had been defined and routinely applied during the study period. According to this protocol, a more radical resection and the TES method providing anterior stability were applied in cases with vertebral body involvement; furthermore, all patients who underwent vertebrectomy with the TES method received neuromonitoring as part of the standard surgical procedure. In the CLDS group, intraoperative neuromonitoring was also employed in patients presenting with spinal cord compression. In cases where only the posterior elements and soft tissues were affected, the CLDS method was preferred. In such cases, stabilisation was applied unilaterally or bilaterally depending on the location of the lesion and the extent of posterior element involvement. Within this scope, bilateral stabilisation was applied in all cases to preserve natural stability in the lumbar region. In the thoracic region, unilateral posterior stabilisation was applied in cases with unilateral involvement, in accordance with the principle of preserving the natural stability of the spine and the minimally invasive surgical approach (Figure 3). There are studies in the literature reporting no significant difference in outcomes between unilateral and bilateral stabilisation in spinal surgery [15]. Given that TES patients all had vertebral body involvement while CLDS patients did not, selection bias may have influenced recurrence outcomes. Preoperative albendazole treatment was not administered due to the need for rapid referral to surgery after diagnosis. All patients received albendazole at a dose of 10 mg/kg/day for at least 6 months postoperatively [12].

### 2.5. Follow-Up and Outcome Measures

All patients were followed up clinically and radiologically by the surgeon who performed the surgery for at least three years. Neurological improvement was defined as an increase in at least one level on the Frankel score 6 months after the operation. Recurrence was determined by the detection of new or progressive lesions consistent with hydatid disease on follow-up MRI.

### 2.6. Statistical Analysis

Data were analysed using IBM SPSS Statistics version 25.0 (IBM Corp., Armonk, NY, USA). Descriptive statistics were presented as medians (minimum–maximum) or means (SD) for continuous variables and as numbers and percentages for categorical variables. The Mann–Whitney U test was used for continuous variables because the data did not follow a normal distribution, while Fisher’s Exact test was used for categorical variables in intergroup comparisons. The researchers considered any *p*-value below 0.05 as statistically significant. Data that were missing were not included in the analysis because the loss rate was less than 5%. The number of operations and the time until recurrence for the patients who experienced recurrence were only provided descriptively for this subgroup.

### 2.7. Ethical Approval and Informed Consent

This study was approved by the Institutional Review Board of Adana City Training and Research Hospital (Meeting No. 90, Decision No. 1596; approval date: 14 October 2021) and conducted in accordance with the principles of the Declaration of Helsinki. According to the institutional protocol followed, all the patients had provided written informed consent at the time of surgery, permitting the use of their anonymised data for scientific research. Because of the retrospective nature of the study, no additional informed consent was required.

## 3. Results

### 3.1. Patient Demographics and Clinical Presentation

The research included twenty-one patients who received surgical treatment for spinal hydatid disease which was confirmed through histopathological examination with thirteen males and eight females and a mean age of 42.6 ± 9.8 years. Total en-bloc spondylectomy was performed on nine patients, while twelve patients underwent cystectomy with a laminectomy and decompression and stabilisation procedures. Baseline demographic and clinical characteristics, including age, gender distribution, symptom duration, presenting symptoms, preoperative neurological status, and hepatic hydatid cyst coexistence, are summarised in Table 1.

All the patients entered the hospital presenting with intense back pain. More than half of the patients in both surgical groups experienced motor weakness, which typically resulted in moderate neurological impairment. Additional presenting symptoms such as sensory disturbances and sphincter dysfunction were also recorded and are detailed in Table 1. The disease mainly affected the thoracic area because seventeen patients had thoracic involvement, while three had lumbar involvement and one had thoracolumbar involvement. (Table 2)

### 3.2. Radiological Findings

**Figure 4:**
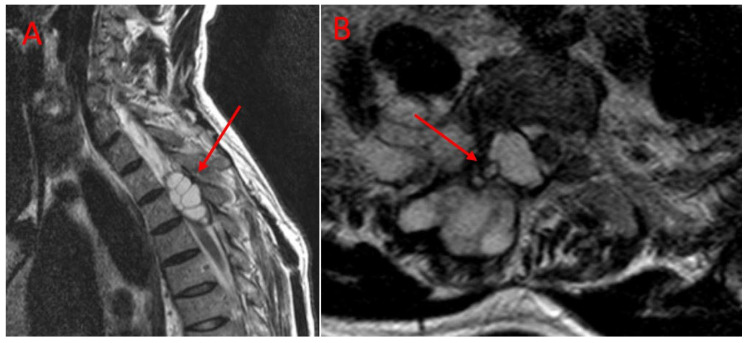
Preoperative T2-weighted MR images of a patient treated with CLDS. (**A**): The arrows indicate multilocular hydatid cysts invading the spinal canal. (**B**): The arrows indicate hydatid cysts (shown from an axial perspective) with a unilateral location extending into the spinal canal and paravertebral muscles.

**Figure 5:**
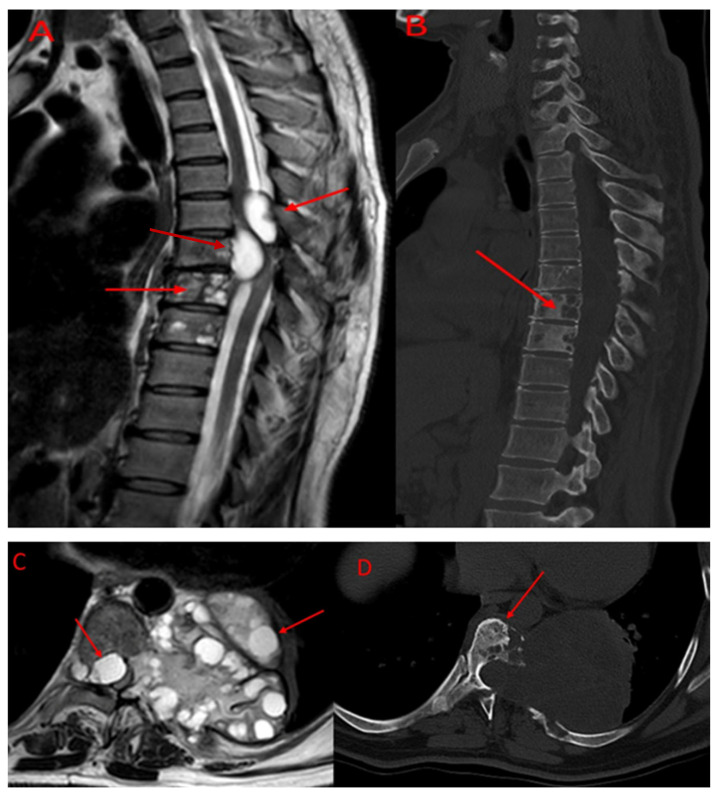
Preoperative imaging of thoracic spinal hydatid cyst. (**A**): Sagittal T2-weighted MRI demonstrating extensive thoracic vertebral involvement by multiloculated hydatid cysts with high signal intensity. Arrows indicate cysts extending into the spinal canal, causing cord compression, as well as multivesicular involvement of the vertebral body. (**B**): Corresponding sagittal CT scan of the same patient. The arrow indicates cortical bone destruction and vertebral involvement. (**C**): Axial section of the MRI shown in Figure 3A. The arrows indicate a cyst compressing the spinal cord and paravertebral cystic involvement. (**D**): Axial CT image corresponding to Figure 3B, showing cortical bone destruction and vertebral involvement. Abbreviations: MRI, magnetic resonance imaging; CT, computed tomography.

Imaging studies revealed that the lesions involved multiple anatomical structures within the vertebral segment. All the patients in the TES group exhibited vertebral body involvement, while the CLDS patients did not present any corpus involvement (*p* < 0.001). Posterior element involvement was universal in the CLDS group and was also common in TES patients. Paravertebral extension was noted in both groups. Major spinal canal invasion (>50%) was observed in both groups (Table 2). Representative MRI and CT images are provided in Figure 4 and Figure 5.

### 3.3. Surgical Outcomes and Perioperative Parameters

We observed major differences in the operational complexity of the surgical methods. TES procedures lasted significantly longer and were associated with greater intraoperative blood loss compared with CLDS, and transfusion was required more frequently in the TES group (Table 3). Dural tears and controlled cyst ruptures were recorded in both groups, and anterior column reconstruction with a titanium cage was performed exclusively in the TES group.

### 3.4. Long-Term Outcomes and Recurrence Patterns

The median follow-up period was 38 months in both groups. Postoperative neurological improvement was observed in both TES and CLDS patients without a significant difference (Table 4). No recurrences occurred in the TES group, whereas recurrence was documented in the CLDS group, and this difference was statistically significant (*p* = 0.004). Recurrent cases required multiple additional surgeries, with the first recurrence typically occurring within the first two years of follow-up. Overall, the total number of operations was higher in the CLDS group. Wound and deep infections, as well as implant failures, were rare in both groups (Table 4).

### 3.5. Laboratory and Serological Findings

No patients received preoperative albendazole owing to urgent surgical requirements in the spinal cord compression setting. All patients completed six months of postoperative albendazole therapy. Eosinophilia, IgG-ELISA positivity, and indirect haemagglutination positivity were observed in both groups without significant differences. Inflammatory markers were mildly elevated, with similar ESR and CRP levels across groups (Table 5).

The clinical results for TES and CLDS match, but their recurrence patterns differ substantially.

## 4. Discussion

Clinical experience and findings from the literature demonstrate that treating spinal hydatid disease through classical surgical decompression methods cannot prevent recurrence, especially when vertebral body invasion occurs [7,16]. The preferred surgical methods for this condition are extensive resection and TES because they extend beyond decompression by removing healthy adjacent tissues. This study emphasises three central points: (a) TES prevents recurrence; (b) CLDS is associated with high recurrence and repeated re-operations; (c) TES perioperative morbidity is acceptable compared with its long-term benefits. This study analyses the long-term follow-up results for spinal hydatid cyst patients who underwent either TES or CLDS performed by the same senior surgeon in an endemic region.

The results for this rare condition are clinically significant due to the surgeon’s extensive experience and consistent follow-up data, despite the limited number of existing studies. In our series, TES was associated with no recurrence, whereas CLDS had a recurrence rate of 58.3%, a finding consistent with previous reports [6,17]. The TES technique takes away both the vertebral body and the back parts, like the lamina and facet joints. This gets rid of all visible and hidden vesicles and microvesicles from the vertebral body, which means a lot of tissue is removed and the risk of recurrence goes down a lot.

In cases where total cyst excision was not possible with either surgical technique, a controlled cyst rupture was performed; this was performed for one patient in the TES group and four patients in the CLDS group. In the literature, a cyst rupture is defined as an important factor that increases the risk of recurrence through the spread of the contents to surrounding tissues [7,16]. Therefore, it is recommended that the procedure be performed under controlled conditions and with appropriate irrigation–aspiration techniques. Indeed, Akhaddar and Boucetta reported no recurrence during a 12-month follow-up period in a case where contamination was prevented using the irrigation–aspiration method [18]. These findings suggest that the en-bloc removal of the vertebral segment containing the cyst, particularly in TES procedures, is effective in minimising the risk of rupture and environmental contamination, thereby reducing recurrence. Additionally, we believe that controlled cyst ruptures, employed in both techniques in cases where the cyst could not be completely removed, contributed to the lower recurrence rate observed in the CLDS group relative to our previous series.

Given the infiltrative nature of hydatid cyst disease, it has been reported that infection can spread from the vertebral body to the posterior elements via the pedicles and that microvesicles can diffuse into the bone [9]. Therefore, limited decompression attempts performed with laminectomy alone cannot prevent the spread of the disease to surrounding tissues, and they increase the risk of recurrence [7,16]. In the TES group, all the patients exhibited vertebral body involvement (100%), whereas no vertebral body involvement was detected in any patient in the CLDS group (*p* < 0.001); posterior element involvement was observed in all patients in the CLDS group (100%). We believe that microvesicles remained latent in the vertebral body in the CLDS group because the vertebral body was not resected, leading to recurrence. In the TES procedure, the en-bloc removal of the diseased vertebral segment significantly reduces the risk of such dissemination and improves surgical efficacy [7]. Herrera and colleagues also recommended completely removing infected bone in lesions involving the vertebral body [11], and in a series of 16 cases treated with TES, no recurrences were observed in three cases [13].

No significant differences were found between the two groups in terms of the number of affected vertebral segments (TES median, 2; CLDS median, 2; *p* = 0.234) (Table 2), suggesting that the surgical technique used may be more decisive in regard to the development of recurrence than the number of segments involved [7]. While no recurrence was observed in the TES group, the high recurrence rate in the CLDS group despite the similar number of segments involved highlights the importance of radical resection.

The mean number of operations for the CLDS patients with recurrence was 2.8, while the total number of operations was 9 in the TES group and 26 in the CLDS group (Table 4). These data are consistent with the rates of multiple surgical requirements reported in the literature. Indeed, the mean number of operations reported in Caglar and colleagues’ series was 2.4 [17], and it was 2.9 in our previous study [6]. In contrast, Sharma et al. reported that only one patient required a second surgery [19].

The median time to recurrence of 14 months (range: 6–28 months) (Table 4) supports the findings of Caglar and colleagues, who reported that recurrence occurred within a mean of 12 months [17]. This duration underscores the clinical importance of conducting close follow-ups with patients, particularly within the first year.

Compared with CLDS, TES was associated with a significantly longer operative time and higher intraoperative blood loss [20,21]. However, these differences were within acceptable ranges and must be weighed against the complete prevention of recurrence. Therefore, the perioperative morbidity of TES appears acceptable when considering the long-term benefits.

The preoperative motor deficit rates in the TES and CLDS groups were 55.6% and 58.3%, respectively. The motor deficit rate in Sioutis and colleagues’ series of 84 patients was 72%, while motor deficits were reported in all patients in Naumov’s series; these data provide support for the notion that spinal hydatid disease may have a widespread effect on motor functions [3,22].

The postoperative neurological recovery rates were 66.7% in the TES group and 58.3% in the CLDS group, with no statistically significant difference between the groups (*p* = 0.698). This result indicates that both surgical techniques have similar degrees of functional efficacy, supporting the comparability of different surgical approaches in spinal hydatid cyst surgery in terms of motor function outcomes.

Spinal canal invasion was detected in 55.6% of the TES group and 66.7% of the CLDS group. The rates show that many patients had significant involvement of the canal. Naumov and colleagues reported spinal canal invasion in all cases of spinal hydatid disease [22], while Majmundar and colleagues reported significant canal invasion in the majority of cases [23], highlighting that although our rates are lower, they still represent substantial canal involvement.

These data emphasise the importance of early diagnosis and timely surgical intervention for patients with spinal hydatid cysts. No preoperative albendazole was used in our series, mainly because rapid surgical planning was initiated without allowing time for medical treatment after the diagnosis was made radiologically.

Although the use of preoperative albendazole has been reported to reduce the risk of recurrence, some series have indicated that this effect is not significant and that surgical delay may adversely affect the neurological prognosis [17]. Therefore, in our clinic, early surgery is preferred over preoperative albendazole in cases with a risk of progressive neurological deficit. All the patients received albendazole treatment for 6 months postoperatively.

This research benefits from the fact that one senior surgeon performed all the surgeries, as this ensured technical consistency in procedures, thereby enhancing the reliability of the results. The patient follow-ups, which were performed regularly and over a long period of time, made it easier to systematically collect detailed postoperative data. We used only thoracic and lumbar spine patients in order to achieve sample homogeneity, while the Frankel score provided standardised functional outcome data for clinical interpretation.

This study has several limitations. This study’s retrospective design introduced the possibility of observational bias in the research methodology. Additionally, the small sample size, which is a result of the rarity of this condition, significantly constrains the statistical power and generalisability of the results. The lack of randomisation requires special attention when making comparisons between groups. This study’s results cannot be applied to all vertebral segments because we excluded patients with cervical and sacral involvement. One surgeon performed all the surgeries. This approach standardised techniques but made it impossible to assess how different surgeons’ experiences would affect the results. The results demonstrate strong surgical technique consistency but require cautious interpretation regarding general applicability. Given the rarity of spinal hydatid disease, large randomised trials are unrealistic; therefore, carefully conducted retrospective series from endemic, high-volume centres remain essential to guide clinical practice.

This study contributes important information to the discussion on surgical effectiveness through its support for the sparse existing literature about the long-term success of the TES method. The optimal duration and dose of preoperative antiparasitic treatment and the long-term functional outcomes of surgical techniques and combined treatment protocols for reducing recurrence rates and the duration of follow-ups remain unclear. These uncertainties highlight the need for further studies, which will require prospective, large-scale, multicentre research to answer these questions.

## 5. Conclusions

The TES approach led to lower recurrence rates than CLDS in spinal hydatid cyst treatment. The equal number of affected vertebral segments between groups indicates that surgical technique extent and radicality might explain the different recurrence rates. These findings demonstrate that radical resection with TES completely prevents recurrence in appropriately selected patients. Extensive surgical procedures can effectively minimise recurrence rates, especially when the cyst affects vertebral bodies. Clinically, TES should be the preferred option in cases with vertebral body involvement, whereas conservative approaches such as CLDS carry a high risk of recurrence and repeated operations.

## Figures and Tables

**Table 1:** Demographic and clinical characteristics of the patients being surgically treated for spinal hydatid disease.

Parameter	TES Group (*n* = 9)	CLDS Group (*n* = 12)	*p* Value
Age, years *	40.5 (20–65)	44.2 (22–68)	0.342 ᵃ
Gender, *n* (%)			0.685 ᵇ
Male	6 (66.7)	7 (58.3)	
Female	3 (33.3)	5 (41.7)	
Symptom duration, months *	8.5 (3–24)	10.2 (4–36)	0.456 ᵃ
Presenting symptoms, *n* (%)			
Back pain	9 (100)	12 (100)	1.000 ᵇ
Motor deficit	5 (55.6)	7 (58.3)	0.898 ᵇ
Sensory deficit	3 (33.3)	5 (41.7)	0.695 ᵇ
Sphincter dysfunction	1 (11.1)	2 (16.7)	0.721 ᵇ
Preoperative Frankel grade, *n* (%)			0.812 ᵃ
A, B	1 (11.1)	2 (16.7)	
C	3 (33.3)	4 (33.3)	
D	4 (44.5)	5 (41.7)	
E	1 (11.1)	1 (8.3)	
Hepatic hydatid cyst	2 (22.2)	3 (25.0)	0.883 ᵇ

* Data are presented as medians (range). ᵃ Mann–Whitney U test, ᵇ Fisher’s exact test. TES: total en-bloc spondylectomy, CLDS: cystectomy, laminectomy, decompression, and stabilisation.

**Table 2:** Radiological and pathological findings in patients with spinal hydatid disease.

Parameter	TES Group (*n* = 9)	CLDS Group (*n* = 12)	*p* Value
Lesion location, *n* (%)			0.754 ᵇ
Thoracic	7 (77.8)	10 (83.3)	
Lumbar	1 (11.1)	2 (16.7)	
Thoracolumbar	1 (11.1)	0 (0)	
Affected vertebrae *	2 (1–3)	2 (1–4)	0.234 ᵃ
Vertebral body involvement	9 (100)	0 (0)	<0.001 ᵇ
Posterior element involvement	7 (77.8)	12 (100)	0.075 ᵇ
Paravertebral extension	3 (33.3)	6 (50.0)	0.449 ᵇ
Spinal canal invasion >50%	5 (55.6)	8 (66.7)	0.606 ᵇ

* Data are presented as medians (ranges). ᵃ Mann–Whitney U test. ᵇ Fisher’s exact test.

**Table 3:** Surgical data and perioperative findings.

Parameter	TES Group (*n* = 9)	CLDS Group (*n* = 12)	*p* Value
Operative time, minutes ^†^	275 ± 30	185 ± 25	<0.001 ᵃ
Intraoperative blood loss, mL ^†^	700 ± 150	450 ± 100	0.002 ᵃ
Transfusion requirement	9 (100)	4 (33.3)	0.002 ᵇ
Controlled cyst rupture	1 (11.1)	4 (33.3)	0.238 ᵇ
Dural injury	1 (11.1)	2 (16.7)	0.222 ᵇ
Anterior column reconstruction			<0.001 ᵇ
Titanium cage	9 (100)	0 (0)	

^†^ Data are presented as means ± standard deviations. ᵃ Mann–Whitney U test. ᵇ Fisher’s exact test.

**Table 4:** Postoperative outcomes and follow-up data.

Parameter	TES Group (*n* = 9)	CLDS Group (*n* = 12)	*p* Value
Follow-up duration, months ^†^	38.1 ± 4.5	38.1 ± 4.5	0.998 ᵃ
Postoperative Frankel grade, *n* (%)			0.445 ᵃ
C	0 (0)	1 (8.3)	
D	3 (33.3)	5 (41.7)	
E	6 (66.7)	6 (50.0)	
Neurological improvement	6 (66.7)	7 (58.3)	0.698 ᵇ
Recurrence	0 (0)	7 (58.3)	0.004 ᵇ
Operations in recurrent cases ^†^	-	2.8 ± 0.6 (2–4)	-
Time to recurrence, months *	-	14 (6–28)	-
Total operations	9	26	-
Wound infection	1 (11.1)	2 (16.7)	0.721 ᵇ
Deep infection	0 (0)	1 (8.3)	0.379 ᵇ
Implant failure	0 (0)	1 (8.3)	0.379 ᵇ

* Data are presented as medians (ranges). ^†^ Data are presented as means ± standard deviations (ranges, where applicable). ᵃ Mann–Whitney U test. ᵇ Fisher’s exact test.

**Table 5:** Medical treatment and laboratory findings.

Parameter	TES Group (*n* = 9)	CLDS Group (*n* = 12)	*p* Value
Postoperative albendazole duration, months *	6 (6–6)	6 (6–6)	1.000 ᵃ
Preoperative eosinophilia (>4%)	4 (44.4)	6 (50.0)	0.801 ᵇ
Preoperative IgG-ELISA positivity	7 (77.8)	9 (75.0)	0.883 ᵇ
Indirect hemagglutination positivity	6 (66.7)	8 (66.7)	1.000 ᵇ
ESR, mm/hour *	28 (12–65)	32 (15–72)	0.567 ᵃ
CRP, mg/L *	12 (3–45)	15 (5–52)	0.445 ᵃ

* Data are presented as medians (ranges). ᵃ Mann–Whitney U test. ᵇ Fisher’s exact test. IgG-ELISA, immunoglobulin G enzyme-linked immunosorbent assay; ESR, erythrocyte sedimentation rate; CRP, C-reactive protein.

## Data Availability

The data presented in this study are available upon request from the corresponding author. The data are not publicly available due to patient privacy restrictions and institutional policies regarding the confidentiality of medical records. Anonymised datasets may be shared with qualified researchers upon reasonable request, subject to approval by the Institutional Review Board of Adana City Training and Research Hospital.

## Abbreviations

The following abbreviations are used in this manuscript:

CLDS	Cystectomy, laminectomy, decompression, and stabilization
CRP	C-reactive protein
CT	Computed tomography
ELISA	Enzyme-linked immunosorbent assay
ESR	Erythrocyte sedimentation rate
IgG	Immunoglobulin G
IHA	Indirect hemagglutination
MRI	Magnetic resonance imaging
SD	Standard deviation
TES	Total en bloc spondylectomy
